# Percutaneous Transhepatic Cholecystoscopic Lithotomy in the Management of Acute Cholecystitis Caused by Gallbladder Stones

**DOI:** 10.1155/DTE.5.19

**Published:** 1998

**Authors:** Shigeki Ohashi

**Affiliations:** 1 Third Department of Internal Medicine Toho University School of Medicine 2-17-6, Meguro-ku Tokyo 153 Japan; 2 88 Higashi sakuragi-chou Aichi-pref. Toyokawa 442-0028 Japan

## Abstract

Percutaneous transhepatic cholecystic drainage (PTCCD) with percutaneous transhepatic cholecystoscopic lithotomy (PTCCSL) were performed in 53 patients with acute cholecystitis caused by gallbladder stones and studied stone removal rates, complications, endoscopic findings, and stone recurrence. The stones were successfully removed in 96% of the patients, and there were no serious complications. The coexistence of cancer was confirmed in three patients, and all cases were accurately diagnosed on the basis of uitrasonographic, endoscopic, and biopsy findings. The mean duration of follow-up after stone removal was 42 months, and the stone recurrence rate was 2.5%. Among the 39 patients followed up for at least 1 year, the gallbladder could be preserved with no evidence of sludge in patients in whom drainage was performed early after the onset of symptoms, those with a normal gallbladder after PTCCSL, and those with normal gallbladder contractility after PTCCSL. Sludge was present in patients with evidence of extensive areas of yellowish white fibers on percutaneous transhepatic cholecystoscopy. If instituted early after the onset of symptoms, PTCCD combined with PTCCSL was considered useful in the treatment of patients with acute cholecystitis associated with gallbladder stones.


*Diagnostic and Therapeutic Endoscopy*, Vol. 5, pp. 19–29
Reprints available directly from the publisher
Photocopying permitted by license only

© 1998 OPA (Overseas Publishers Association) N.V.
Published by license under
the Harwood Academic Publishers imprint,
part of The Gordon and Breach Publishing Group.
Printed in Singapore.

### Percutaneous Transhepatic Cholecystoscopic Lithotomy in the Management of Acute Cholecystitis Caused by Gallbladder Stones

SHIGEKI OHASHI *

*Third Department of Internal Medicine, Toho University School of Medicine, 2-17-6, Meguro-ku, Tokyo 153, Japan*

*(Received 2 April 1997; In final form 21 November 1997)*

**Percutaneous transhepatic cholecystic drainage (PTCCD) with percutaneous transhepatic cholecystoscopic lithotomy (PTCCSL) were performed in 53 patients with acute cholecystitis caused by gallbladder stones and studied stone removal rates, complications, endoscopic findings, and stone recurrence. The stones were successfully removed in 96% of the patients, and there were no serious complications. The coexistence of cancer was confirmed in three patients, and all cases were accurately diagnosed on the basis of ultrasonographic, endoscopic, and biopsy findings. The mean duration of follow-up after stone removal was 42 months, and the stone recurrence rate was 2.5%. Among the 39 patients followed up for at least 1 year, the gallbladder could be preserved with no evidence of sludge in patients in whom drainage was performed early after the onset of symptoms, those with a normal gallbladder after PTCCSL, and those with normal gallbladder contractility after PTCCSL. Sludge was present in patients with evidence of extensive areas of yellowish white fibers on percutaneous transhepatic cholecystoscopy. If instituted early after the onset of symptoms, PTCCD combined with PTCCSL was considered useful in the treatment of patients with acute cholecystitis associated with gallbladder stones.**

*Keywords:* Gallbladder stone, Acute cholecystitis, Percutaneous transhepatic cholecystoscopy, Lithotomy, Stone recurrence

### INTRODUCTION

Percutaneous transhepatic cholecystic drainage (PTCCD) is useful in the treatment of acute cholecystitis caused by gallbladder stones [1]. After PTCCD, percutaneous transhepatic cholecystoscopy (PTCCS), via the fistula used for drainage, with percutaneous transhepatic cholecystoscopic lithotomy (PTCCSL) has been reported to be a useful procedure in elderly patients and high-risk patients with serious underlying disease [1,2]. However, experience with PTCCSL in patients

* Address for correspondence: 88 Higashi sakuragi-chou, Toyokawa-city, Aichi-pref., 442-0028, Japan. Tel.: (0533)-86-2354. Fax: (0533)-85-2728.

with acute cholecystitis and gallbladder stones is limited, and no study has evaluated the safety and efficacy of this procedure in detail. To evaluate the potential of PTCCSL as a procedure for lithotomy and to assess its ability to preserve gallbladder function in patients with acute cholecystitis, this study was undertaken.

### SUBJECTS AND METHODS

The study group comprised 53 patients with acute cholecystitis who underwent lithotomy by PTCCSL between October 1987 and February 1994. All patients presented with spontaneous pain and tenderness of the right hypochondrium or epigastrium and had a fever of at least 37°C and leukocytosis, as defined by a leukocyte count of $9 \times 10^3$ cells/μl or more. Ultrasonographic examination revealed evidence of an enlarged gallbladder, thickening of the gallbladder wall, or the presence of sludge inside the gallbladder. PTCCD was performed for a diagnosis of acute cholecystitis caused by gallbladder stones. The patients were given a detailed explanation of the procedure before PTCCD and PTCCSL, and informed consent was obtained before performing either procedure.

An 18 G needle was used for PTCCD. In principle, the needle was inserted transhepatically via the gallbladder bed, under ultrasonographic guidance. PTCCD was performed with the use of a 8 Fr drainage tube equipped with a balloon. The fistula was dilatated by insertion of a 16 or 18 Fr tube 3–7 days after insertion of the 8 Fr tube, or a 16 or 18 Fr tube was inserted on the same day instead of the 8 Fr drainage tube. Dilatation was performed with the use of a coaxial dilator (PTC-Ichikawa-A-16 or PTC-Ichikawa-A-18, Cook Co., Ltd., Bloomington, USA) as described by Ichikawa *et al.* [3]. PTCCS was performed 1 week after fistula dilatation. For endoscopy, models CHF-P10, CHF-P20Q, and CHF-T20 endoscopes (Olympus Optical Co., Ltd., Tokyo, Japan) were used. The gallstones were crushed with an electrohydraulic lithotriptor (EHL), and the stone fragments were removed with a basket catheter or a lithotomy forceps under endoscopic guidance. About 3 days after stone removal, PTCCS was performed to confirm that no stones remained. The absence of stone fragments was confirmed by conventional observation and after the application of 0.5% methylene blue solution. Endoscopic examinations were able to be performed in 49 patients; they were not possible in 3 patients with concurrent gallbladder carcinoma and in 1 patient in whom bleeding precluded observation of the mucosa. The basic pattern of the gallbladder mucosa, defined as that accounting for two-thirds of the entire mucosal surface, was classified as reticular (Fig. 1), granular (Fig. 2), flat (Fig. 3), or deteriorated pattern (DET) (Fig. 4).

In 40 patients the presence or absence of stone recurrence or sludge accumulation was confirmed by ultrasonography 4 weeks, 1 and more year after removal of the biliary drainage tube. Follow-up examinations were not possible in 13 patients: 3 because of gallbladder carcinoma, 4 who underwent cholecystectomy at the time of laparotomy due to another disease, and 6 who were lost to follow-up after PTCCSL. At follow-up, the maximum transverse area of the gallbladder was estimated by the trace technique before and 15 min after the intramuscular injection of 0.2 mg/m$^2$ ceruletide diethylamine. Gallbladder contractility was calculated by dividing the transverse area of the gallbladder after intramuscular injection by that before intramuscular injection. The patients were also divided into those with evidence of gallbladder sludge at the time of follow-up (sludge-positive group) and those with no evidence of gallbladder sludge (sludge-negative group) to study if PTCCSL was effective in preventing stone recurrence. The time from the onset of acute cholecystitis was calculated from the day on which fever or abdominal pain developed, as ascertained by interview. Stone size was measured on PTCCD films taken before lithotomy. The stones removed at the time of PTCCSL were analyzed to determine their composition. In principle, after PTCCSL the patients were given 600 mg/day of UDCA to prevent stone recurrence. The chi-square test was used for statistical

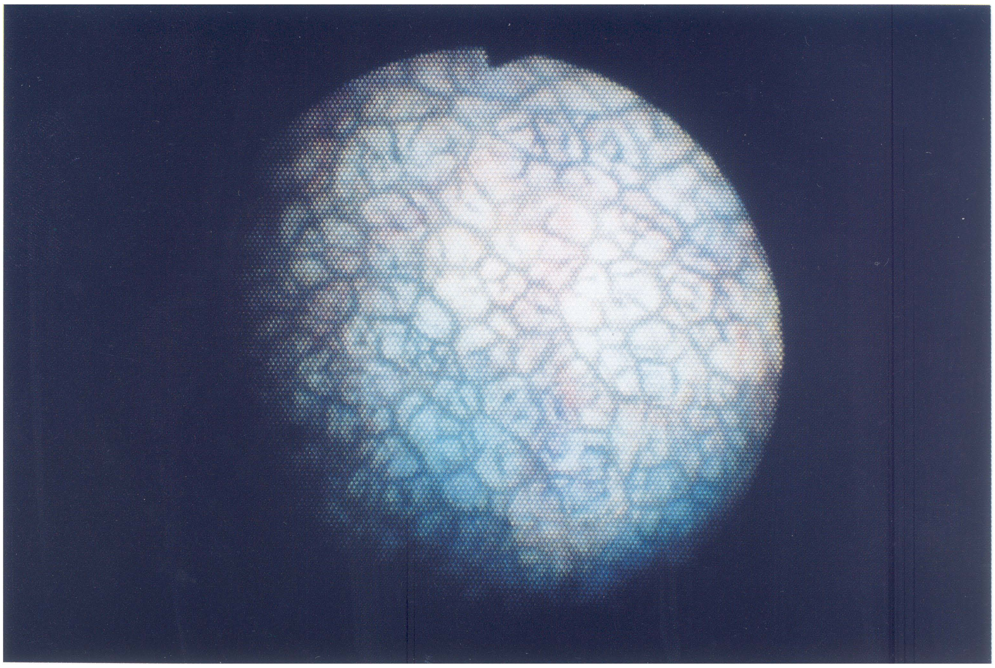

FIGURE 1 Endoscopic findings after dyeing with methylene-blue solution. There was a reticular structure on the mucosa, associated with mild changes suggesting acute cholecystitis.

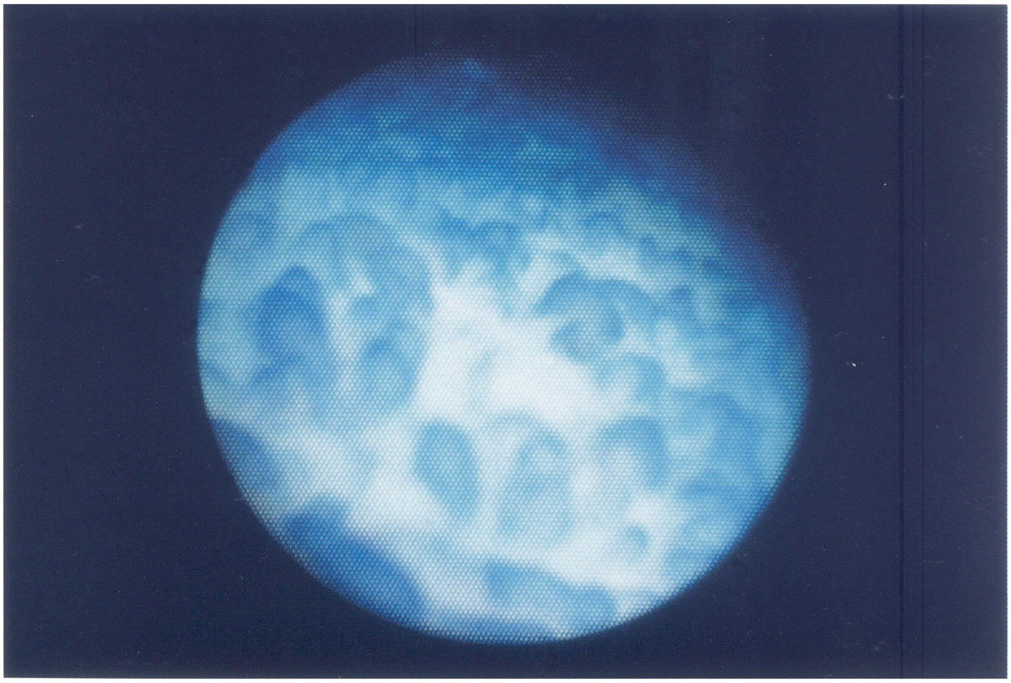

FIGURE 2 Endoscopic findings after dyeing with methylene-blue solution. The mucosa had a homogenous, finely granular appearance, without fusion between the granules.

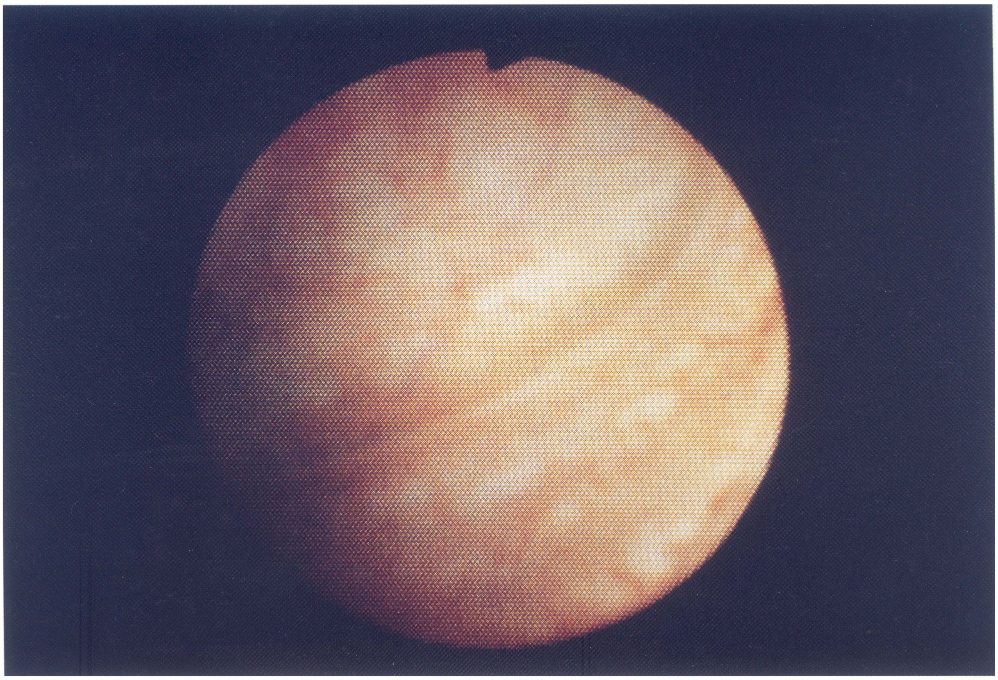

FIGURE 3 Endoscopic findings. The mucosa had lost its reticular structure and resembled atrophic gastritis.

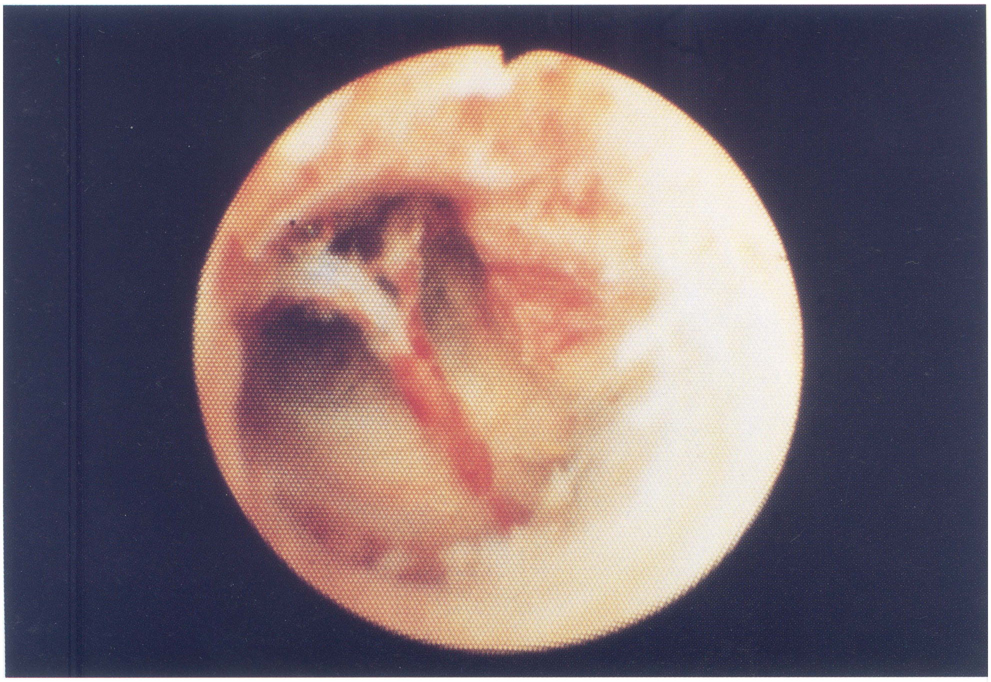

FIGURE 4 Endoscopic findings in a case of necrotic cholecystitis. Rough deteriorated yellowish white fibers covered the inside of the lumen, which had reddish columnar mucosa with severe mucosal necrosis. The distensibility of the wall was reduced.

analysis. $P$ values of less than 0.05 were considered to indicate statistical significance.

### RESULTS

### 1. Classification of Stones and Clinical Signs and Symptoms of Acute Cholecystitis

Twenty-two men and 31 women with a mean age of 64.2 years (range, 32–89) were studied. Stones were analyzed in 49 of the 53 patients. Twenty-nine patients had cholesterol stones, and 20 had bilirubin stones. Before lithotomy the mean maximum stone diameter was 14.4 mm; 36 patients (68%) had stones with maximum diameters of 10 mm or more. The mean period from onset of symptoms to PTCCD was 5.1 days, and the mean period from admission until PTCCD was 2.1 days. A total of 44 patients (83%) underwent PTCCD within the first hospital day. Before PTCCD all patients had abdominal pain, 86% had fever, and 85% had leukocytosis. Abdominal pain resolved promptly after PTCCD, followed by the resolution of fever and leukocytosis, in that order. All signs and symptoms of inflammation disappeared within 5 days, and the clinical condition of all patients improved.

### 2. Outcome of PTCCSL

Lithotomy by PTCCSL was performed from one to six times (mean, 2.5 times per patient). Forty-two patients underwent PTCCSL three times or less, and the stones could be completely pulverized in 51 patients (96%). Lithotripsy was not possible in 2 patients because of bleeding (1) or cystic duct injury (1). In this latter patient, a small amount of contrast media had leaked out from the cystic duct on imaging examinations during PTCCSL. Although this case could be managed conservatively, cholecystectomy was performed at the patient's request. The patient with gallbladder bleeding had renal failure associated with a hemorrhagic diathesis. Exudative bleeding occurred, and hemostasis was performed conservatively. There were no other serious complications.

### 3. PTCCS Findings after Lithotripsy

The basic pattern of the mucosa was classifiable in 49 patients. It was reticular in 28 patients (58%), granular in 11 patients (23.1%), flat in 6 patients (12.6%), and DET in 4 patients (6.3%). In the DET cases, the lumen appeared to contain yellowish white fibrous material, the distensibility of the wall was reduced.

### 4. Recurrent Stones after PTCCSL

Among 40 patients in whom the gallbladder was preserved who were able to be followed up, new stone occurred in 1 patient (2.5%). She had bilirubin stones at the initial episode and at recurrence. Gallbladder contractility decreased to 73% after the initial session of PTCCSL. Stone recurrence was confirmed on abdominal ultrasonography 5 months after lithotomy. The patient was asymptomatic and followed up without any treatment. After 10 months the patient experienced abdominal pain, and cholecystitis developed. PTCCSL was repeated at the patient's request. Subsequently, there have been no signs or symptoms of recurrence.

### 5. Sludge at Follow-up

The long-term possibility of stone recurrence was forecast on the basis of the presence or absence of sludge.

#### *(1) Clinical Demographic Characteristics (Table 1)*

The follow-up period was 46 months in the sludge-positive group and 40 months in the sludge-negative group. This difference was not significant. There were also no distinct differences in male : female ratio, mean age, stone composition, or maximal stone diameter. There was a

TABLE I Clinical demographic characteristics

| Sludge | Positive group$_{n=15}$ | Negative group$_{n=24}$ |
|---|---|---|
| Mean duration of follow up (month) | 46 ± 15 | 40 ± 14 |
| Sex (male : female) | 5 : 10 | 10 : 14 |
| Stone | | |
| Component (CH : BI) | 7 : 8 | 12 : 12 |
| Mean maximum in diameter (mm) | 13.1 ± 8.0 | 15.5 ± 8.8 |
| Choleretic agent after PTCCSL | 11/15 | 23/24 |

CH: cholesterol stone, BI: bilirubinate stone, PTCCSL: percutaneous transhepatic cholecystoscopic lithotomy.

TABLE II Clinical signs, symptoms and course

| Sludge | Positive group$_{n=15}$ | Negative group$_{n=24}$ |
|---|---|---|
| *Before PTCCD* | | |
| Temperature (°C) | 37.3 ± 0.5 | 37.8 ± 1.0 |
| Leukocyte count | 12700 ± 5900 | 14300 ± 6500 |
| Mean period from onset until PTCCD (day) | 10.5 ± 12.3* | 2.56 ± 2.57* |
| *Mean period until improvement after PTCCD (day)* | | |
| Abdominal pain | 0.4 ± 0.8 | 0.1 ± 0.3 |
| Fever | 1.5 ± 1.3 | 1.2 ± 1.2 |
| Leukocytosis | 6.1 ± 5.4 | 5.0 ± 5.9 |
| Mean session of PTCCSL | 2.6 ± 1.6 | 2.3 ± 1.1 |

PTCCD: percutaneous transhepatic cholecystic drainage, PTCCSL: percutaneous transhepatic cholecystoscopic lithotomy.
*$p < 0.05$.

trend toward a lower proportion of patients receiving choleretic agents in the sludge-positive group, but the difference was not statistically significant. The clinical demographic characteristics of the patients in the sludge-positive group were thus similar to those in the sludge-negative group.

### (2) *Clinical Signs and Symptoms and Course (Table II)*

There were no differences between the groups in body temperature or leukocyte count before PTCCD, and the inflammatory response was considered similar. The mean period from onset until PTCCD was 2.5 days in the sludge-negative group, as compared with more than 10 days in the sludge-positive group. Clearly, PTCCD was started earlier in the sludge-negative group. There was no distinct difference between the groups in the improvement of symptoms after PTCCD. The mean number of attempts at PTCCSL was 2.6 times in the sludge-positive group and 2.3 times in the sludge-negative group.

### (3) *Ultrasonographic Findings (Table III)*

Before PTCCD evidence of thickening of the gallbladder wall was found in all patients in both groups. A three-layered structure of the wall was present in 79% and 73% and a hypoechoic area around the gallbladder in 20% and 25% of the sludge-negative group and the sludge-positive group, respectively. These differences were not significant.

The cross-sectional of the gallbladder 4 weeks after PTCCSL was normal in the sludge-negative group, while it decreased to a mean value of 628 $mm^3$ in the sludge-positive group. This difference was significant. The mean rate of

TABLE III Ultrasonographic findings of gallbladder

| Sludge | Positive group$_{n=15}$ | Negative group$_{n=24}$ |
|---|---|---|
| *Before PTCCD* | | |
| Thickening of wall (%) | 15 (100) | 24 (100) |
| Three layered structure | 11 (73) | 19 (79) |
| Sludge | 12 (80) | 18 (75) |
| HEA | 3 (20) | 6 (25) |
| *After PTCCSL* | | |
| Mean thickening of wall (mm) | 4.1 ± 1.7 | 3.4 ± 1.2 |
| Mean value (mm$^3$) | 628 ± 348* | 1021 ± 380* |
| Mean rate of contractility (%) | 77.3 ± 22.1* | 51.1 ± 18.0* |

HEA: hypoechoic area around the gallbladder, PTCCD: percutaneous transhepatic cholecystic drainage, PTCCSL: percutaneous transhepatic cholecystoscopic lithotomy.
*$p < 0.01$.

TABLE IV Endoscopic findings

| Sludge | Positive group$_{n=15}$ (%) | Negative group$_{n=24}$ (%) |
|---|---|---|
| Reticular | 7 (46) | 16 (42) |
| Granular | 3 (20) | 5 (13) |
| Flat | 1 (7) | 4 (17) |
| DET | 4 (27) | 0 (0) |

DET: deteriorated.

contractility, calculated by dividing the gallbladder area after intramuscular injection of ceruletide diethylamine by that before injection, was 51% in the sludge-negative group, indicating normal contractility, and 77% in the sludge-positive group, indicating decreased contractility. This difference was significant.

### (*4*) *Endoscopic Findings* (*Table IV*)

A DET pattern was found only in the sludge-positive group. There were other significant differences between the groups.

### DISCUSSION

If not treated properly, acute cholecystitis can rapidly progress to a serious infection. Emergency cholecystectomy or PTCCD with antibiotic treatment has been recommended for acute cholecystitis [4–6]. In recent years, laparoscopic cholecystectomy (LC) has gradually replaced conventional open surgery in patients with acute cholecystitis, and some centers perform LC as soon as the diagnosis is established [7]. Preoperatively, however, information on the biliary tract is often lacking in patients with acute cholecystitis, and the induction of pneumoperitoneum in the presence of acute cholecystitis carries the risk of septicemia. Some reports have therefore recommended against LC in patients with acute cholecystitis [8]. Other reports have suggested that cholecystectomy causes active proliferation of the colonic mucosa and increases the risk of carcinogenesis [9]. In place of cholecystectomy, PTCCSL thus holds much promise as a procedure for stone removal and the treatment of acute cholecystitis.

After PTCCD, PTCCSL has been reported to be optimally suited for the treatment of elderly patients and high-risk patients who cannot tolerate open surgery [1]. The authors place a PTCCD tube in patients with acute cholecystitis to permit drainage. This promptly improves inflammation, permits examination of the biliary tract, and facilitates formulation of the subsequent therapeutic policy. I therefore believe that PTCCD should be performed as soon as a diagnosis of acute cholecystitis has been established, without an intervening period of conservative therapy.

The development of ultrasonic equipment and the availability of improved puncture needles and indwelling tubes have enabled an enlarged, acutely inflamed gallbladder to be punctured reliably. Tube dislodgment, a complication reported early after the advent of PTCCD, did not occur in any of our patients, Eric *et al.*, similarly reported that tube dislodgment occurred in only 1 of 127 patients who underwent percutaneous gallbladder puncture [10]. PTCCD is therefore considered to be optimally suited for the treatment of acute cholecystitis. In addition, since the fistula closes about 1 week after puncture, the stones could be removed under PTCCS guidance in all patients [11]. Complications caused by PTCCSL occurred in a total of 2 patients (3.8%): one with gallbladder bleeding and one with injury to the cystic duct. Ichikawa *et al.* reported gallbladder bleeding in three patients (5.8%), but there were no other serious complications; treatment was therefore able to be performed conservatively [1]. EHL is the most reliable treatment for lithotomy under endoscopic guidance. PTCCSL is most difficult to perform when the stone leaves the neck of the gallbladder and enters the spiral part of the cystic duct, precluding a direct view on endoscopy. The injury to the cystic duct described above occurred while attempting to remove by EHL a stone from the cystic duct. Endoscopic procedures are especially difficult to perform in the narrow region from the neck of the gallbladder to the convoluted part of the cystic duct; EHL should therefore be performed only when a direct view of the stone can be obtained. Since the stone is incarcerated, the injured gallbladder mucosa is fragile. Lithotripsy must be performed with extreme care. With the exception of this case, stone removal was successfully accomplished endoscopically in all other patients. Lithotomy was possible under direct vision in all patients, but the concurrent use of extracorporeal shock-wave lithotripsy or direct litholysis is useful at sites unable to be viewed directly.

We had expected to observe a wide range of endoscopic findings on PTCCS because of changes of the gallbladder mucosa caused by the long-term presence of one or more stones and changes due to acute cholecystitis. The basic findings of the mucosa were therefore evaluated separately from other findings. Watanabe *et al.* classified the surface pattern of the gallbladder mucosa as reticular, funicular, granular, papillary, or lobular according to resected specimens of normal mucosa, metaplastic mucosa, and carcinoma [12]. I modified this system and classified endoscopic findings of the gallbladder mucosa in acute cholecystitis as reticular, granular, flat, or DET. About 56% of the cases had a reticular mucosa, and there were few cases with a flat or granular mucosa; most patients had nearly normal endoscopic findings. Watanabe *et al.* reported that pathological evidence of a reticular pattern of the mucosa is seen in normal tissue or metaplastic tissue [12]. Since the reticular mucosa in this study was in patients with gallbladder stones, the gallbladder mucosa in this series most likely had some type of metaplastic changes. In the patients who were followed up, the granular mucosa had a homogenous, finely granular appearance, and there was no evidence of fusion between granules or of asymmetry. In patients with gallbladder carcinoma, we have found papillary and nodular patterns of the mucosa combined with a granular appearance. Even in areas of granular mucosa, the granules were irregularly shaped and showed irregular areas of redness. Cancer could be diagnosed on the basis of these findings, as reported by Inui *et al.* [13]. The flat mucosa has flattened reticular sites and resembles the atrophic mucosa seen in gastritis. Since there are also some areas of funicular mucosa, the flat mucosa is similar to the funicular and the flat mucosa described by Watanabe *et al.* and it is thought to reflect deterioration of the gallbladder mucosa. A yellowish white, thick unstructured filamentous structure with no appreciable signs of a DET pattern of the gallbladder mucosa was seen in four patients. These patients were left untreated despite remarkable inflammation for a prolonged period or appeared to have emphysematous cholecystitis, leading to the development of very severe mucosal necrosis.

Prior reports have described the concurrent presence of acute cholecystitis and gallbladder carcinoma [14]. This series also included three patients (5.6%) who concurrently had cancer. In patients with cholecystitis the decision whether or not to preserve the gallbladder depends largely on the presence or absence of cancer. During the acute phase of acute cholecystitis, sludge often accumulates in the gallbladder, and the properties of the mucosa are indistinct on ultrasonographic examination. About 74% of early carcinomas of the gallbladder have been reported to be superficial carcinomas [12]. Since diagnosis is extremely difficult, carcinomas are often found accidently on pathological examination of the resected gallbladder, particularly when there was evidence of a protruding lesion on extracorporeal ultrasonography. With the use of PTCCD as described above, acute inflammation can be confined to a minimum, and accurate diagnosis is possible by biopsy and examination of the mucosa by PTCCS and by cytological diagnosis of the bile obtained from the drainage tube.

In patients who concurrently have cancer, PTCCS may cause metastasis via the drainage fistula [15]. I treat the fistula with alcohol in patients with cancer. Histological studies were performed in one patient who subsequently underwent surgery, but there was no evidence of metastasis. Even in patients who were unable to undergo surgery, there was no evidence of invasion to the area around the fistula on CT or extracorporeal ultrasonography.

After PTCCSL, patients are given 600 mg/day of UDCA to prevent stone recurrence. A previous study has reported that about 1 week after the termination of choleretic agents the degree of cholesterol saturation returns to the pre-treatment level, which increases the risk of stone recurrence [16]. The continuous administration of oral gallstone-dissolving agents is considered effective in preventing stone recurrence. Dowling *et al.* found that the 12-month rate of recurrence with oral gallstone dissolving agents ranged from 10% to 15% [17]. During follow-up, I had only one case (2.5%) of recurrence, which is clearly low as compared with previous reports. This patient had a bilirubin stone, and gallbladder contractility was low (73%). Acute cholecystitis recurred after about 10 months. PTCCSL was repeated at the patient's request, and the stone was removed. In such patients who have distinct evidence of low gallbladder function, the preservation of function should not be the ultimate goal of treatment, but this case demonstrates the high acceptance of PTCCSL.

The mean period of follow-up after PTCCSL in 40 patients was only about 3.5 years. The presence of sludge in the gallbladder was an important factor in predicting the long-term outcome in these patients. Bernard *et al.* studied a series of patients with decreased gallbladder function who received total parenteral nutrition for 6 months [18]. During follow-up, gallstones formed only in patients with sludge in the gallbladder; there was no evidence of stone formation in patients with no sludge. I divided 39 of patients, excluding the one with stone recurrence after PTCCSL, into a sludge-positive group and a sludge-negative group on the basis of the presence or absence of ultrasonographic evidence of sludge and compared their clinical demographic characteristics, clinical signs and symptoms of acute cholecystitis, ultrasonographic findings of the abdomen, and endoscopic findings. The mean period of follow-up was similar in the sludge-positive group (46 months) and the sludge-negative group (40 months). There were no significant differences between the groups in other clinical characteristics or in the type of gallstone or the maximum stone diameter. The proportion of patients receiving choleretic agents was slightly, but not significantly, higher in the sludge-negative group, and there was no significant differences in any clinical characteristic.

As for the clinical signs and symptoms of acute cholecystitis, there were no significant differences between the groups in body temperature or leukocyte count before PTCCD, and the severity of inflammation was considered similar. There were also no distinct differences between the groups with respect to the improvement in symptoms after PTCCD or the response to treatment. In both groups PTCCSL was performed three times or less in the majority of patients. The only difference noted was that the period from the onset of symptoms until PTCCD was 10 days or more in the sludge-positive group, as compared with 2.5 days or less in the sludge-negative group; treatment by PTCCD was clearly started earlier in the sludge-negative group. Acute cholecystitis is characterized by acute obstructive cholecystopathy caused by obstruction of the cystic duct or the neck of the gallbladder. In studies of the time course of acute cholecystitis, Mutoh *et al.* found that congestion and edema of the gallbladder wall develops 2–4 days after stone impaction and obstruction [19]. The gallbladder tissue is initially preserved, but continued obstruction leads to increased luminal pressure within the gallbladder and to circulatory failure, which results in tissue necrosis. After about 5 days, abscess formation occurs inside the gallbladder as well as around the gallbladder. In the present study, PTCCD was performed immediately after diagnosis, and the mean period from initial examination until PTCCD was short, especially in the sludge-negative group. Given that circulatory failure due to obstruction of the cystic duct is the main feature of acute cholecystitis, in patients in whom circulatory failure responded to early drainage, necrosis of the gallbladder wall could also be avoided and the function of the gallbladder could be preserved. This was apparently responsible for the better outcome in terms of gallbladder function the sludge-negative group, in which drainage was performed earlier than in the sludge-positive group.

When ultrasonograms were compared, there was no distinct difference in findings before PTCCD. However, on follow-up examination after PTCCSL, the mean area had decreased to 628 $mm^3$ in the sludge-positive group, but a normal area of 1021 $mm^3$ was maintained in the sludge-negative group. The mean area after the intramuscular injection of ceruletide diethylamine had decreased to 77.3% of the pre-injection area in the sludge-positive group, whereas the value remained within the normal range of 51.1% in the sludge-negative group. As for ultrasonographic findings of acute cholecystitis, Guy *et al.* reported that the hyperechoic region of the gallbladder wall indicated are as of inflammatory edema and that the hyperechoic region in the early stage improved in response to conservative therapy [20]. On the other hand, similar hyperechoic regions have been reported to indicate abscess formation, and the surrounding hyperechoic areas pericystic abscess formation [21–23]. The presence of these ultrasonographic findings indicates a high risk of gangrenous cholecystitis. In the present study, good function was preserved in some patients with these ultrasonographic findings. The presence of such findings alone therefore does not provide an adequate basis for the prediction of gallbladder function after PTCCSL. Further studies analyzing the qualitative and quantitative characteristics of hyperechoic areas around the gallbladder are required to establish the diagnostic value of ultrasonography.

On endoscopic examination, the incidences of reticular, granular, and flat patterns of the gallbladder were similar in both the sludge-positive group and the sludge-negative group. However, a DET pattern, indicating epithelial loss and the presence of extensive areas of yellowish white fibers covering the inside of the lumen, was found only in the sludge-positive group. These patients also had decreased contractility, and endoscopic evidence of a DET pattern is considered characteristic of severe cholecystitis with virtually complete loss of gallbladder function. Since such cases progress gradually to an atrophic gallbladder, a DET pattern is regarded to correspond to acute phlegmon type or abscess type, as described by Itoi *et al.* [24]. These endoscopic findings are considered to reflect necrosis of all layers of the gallbladder wall. Because the gallbladder becomes atrophic in these patients, there is a low risk of stone recurrence, and the long-term outcome is generally good.

The results of this study indicate that the drainage in patients with acute cholecystitis can promptly resolve inflammation. In addition, endoscopic lithotomy can be performed via the drainage fistula to remove gallstones. The presence or absence of cancer can be concurrently evaluated. According to this study, gallbladder function did not recover satisfactorily in the patient who had evidence of progression to gangrenous cholecystitis on ultrasonographic and endoscopic examinations. Therefore in patients with stone recurrence or repeated episodes of cholecystitis, cholecystectomy will be the treatment of choice. However, PTCCSL is considered useful in both elderly patients with serious concurrent conditions as well as in younger patients. If performed promptly after the onset of acute cholecystitis, PTCCSL is a safe technique that can provide contribute significantly to the preservation of gallbladder function.

*Acknowledgments*

The author is grateful to Prof. Tetsu Yamaguchi, Prof. Yoshihiro Sakai, and Dr. Iruru Maetani, Lecturer, of the Third Department of Internal Medicine, Toho University, for their guidance in this study and review of the manuscript. The author also sincerely appreciate the help and cooperation of colleagues in the Third Department of Internal Medicine, Toho University.

### *References*

[1] Ichikawa, K., Ichikawa, M., Takahashi, S. *et al.* Percutaneous transhepatic cholecystoscopy (PTCCS) for the nonsurgical treatment of acute cholecystitis. *J. Biliary Tract and Pancreas* 1992; **13**: 743–750 (in Japanese).

[2] Inui, K., Nakazawa, S., Naito, Y. *et al.* Nonsurgical treatment of cholecystolithiasis with percutaneous transhepatic cholecystoscopy. *Am. J. Gastroenterology* 1988; **83**: 1124–1127.

[3] Ichkawa, K., Nakazawa, S., Naitou, Y. *et al.* Diagnosis of protuberant lesions of the gallbladder by PTCCS and PTDCC. *J. Biliary Tract and Pancreas* 1985; **6**: 39–44 (in Japanese).

[4] Addison, N.V. and Fianan, P.J. Urgent and early cholecystectomy for acute gallbladder disease. *Br. J. Surg.* 1988; **75**: 141–143.

[5] Linden, W. and Edlund, G. Early versus delayed cholecystectomy. The effect of a change in management. *Br. J. Surg.* 1981; **68**: 753–757.

[6] Mcsherry, C.K. Cholecystectomy. The gold standard. *Am. J. Surg.* 1989; **158**: 174–178.

[7] Flowers, J.L., Bailey, R.W., Scovill, W.A. *et al.* The baltimore experience with laparoscopic management of acute cholecystitis. *Am. J. Surg.* 1991; **161**: 388–392.

[8] Idezuki, Y. and Otomo, Y. Laparoscopic cholecystectomy – Present status and prospect in future. *J. Biliary Tract and Pancreas* 1992; **13**: 1–5 (In Japanese).

[9] Soltero, E., Cruz, N.I., Nazario, C.M. *et al.* Cholecystectomy and right colon cancer in Puerto Rico. *Cancer* 1990; **66**: 2249–2252.

[10] Eric, V., Horacio, B., Brian, W. *et al.* Percutaneous gallbladder puncture and cholecystostomy. Result, complications, and caveats for safety. *Radiology* 1992; **183**: 167–170.

[11] Maetani, I., Ohashi, S., Ogawa, S. *et al.* Percutaneous cholecystolithotomy for treatment of acute cholecystitis using PTCCS. *Diagnostic Imaging of the Abdomen* 1994; **14**: 224–231 (in Japanese).

[12] Watanabe, H., Kijima, H., Uchida, K. *et al.* Definition and morphological characteristics of early carcinoma of the gallbladder. *Stomach and Intestine* 1986; **21**: 483–495 (in Japanese).

[13] Inui, K., Nakazawa, S., Yoshio, J. *et al.* Cholecystoscopy and endoscopic ultrasonography. *J. Biliary Tract and Pancreas* 1992; **13**: 143–148 (in Japanese).

[14] Douglas, A. Person: Carcinoma of the gallbladder presenting as acute cholecystitis and leading to a missed clinical and pathologic diagnosis. *Am. J. Surg.* 1964; **108**: 95–97.

[15] Juan, A.O., Ernest, J.R., David, B.F. *et al.* Extension of neoplasm along the tract of a transhepatic tube. *Am. J. Radiology* 1980; **135**: 841–842.

[16] Iser, J.H., Murphy, G.M. and Dowling, H. Speed of change in bilialy lipids and bile acids with choledeoxycholic acid. Is intermittent therapy feasible? *Gut* 1977; **18**: 7–15.

[17] Dowling, R.H., Gleeson, D.C., Hood, K.A. *et al.* Gallstone recurrence and postdissolution management. Bile acids and the Liver. MPT press limited. 1986: 355–367.

[18] Bernard, M., Chistian, B., Francis, K. *et al.* Dose total parenteral nutrition induce gallbladder sludge formation and lithiasis. *Gasteroenterology* 1983; **84**: 1012–1019.

[19] Mutou, Y., Uchida, M., Waki, S. *et al.* Clinicophathological study of obstructive cholecystpathy – Relationship of gallbladder lesion to clinical symptom. *J. Japan Surgical Society* 1981; **14**: 672–677 (in Japanese).

[20] Guy, J.F., Marc, C., Albert, L. *et al.* Gallbladder wall sonolucency in acute cholecystitis. *Radiology* 1979; **133**: 429–433.

[21] Takada, T., Yasuda, H., Uchiyama, K. *et al.* Pericholecystic abscess-Classification of US findings to determine the proper therapy. *Radiology* 1989; **172**: 693–697.

[22] Bergman, A.B., Neiman, H.L. and Kraut, B. Ultrasonographic evaluation of pericholecystic abscesses. *A. J. R.* 1979; **132**: 201–203.

[23] Crade, M., Taylor, K.J.W., Rosenfield, A.T. *et al.* Ultrasonic imaging of pericholecystitic inflammation. *J. A. M. A.* 1980; **15**: 708–709.

[24] Itoi, T., Watanabe, H., Takei, K. *et al.* Pathology of the acute cholecystitis – Its classification and transition of gross type and its differentiation from carcinoma. *Diagnostic Imaging of the Abdomen* 1994; **14**: 143–156 (in Japanese).

